# PLD4 Is Involved in Phagocytosis of Microglia: Expression and Localization Changes of PLD4 Are Correlated with Activation State of Microglia

**DOI:** 10.1371/journal.pone.0027544

**Published:** 2011-11-15

**Authors:** Yoshinori Otani, Yoshihide Yamaguchi, Yumi Sato, Teiichi Furuichi, Kazuhiro Ikenaka, Hiroshi Kitani, Hiroko Baba

**Affiliations:** 1 Department of Molecular Neurobiology, School of Pharmacy, Tokyo University of Pharmacy and Life Sciences, Hachioji, Tokyo, Japan; 2 Laboratoy for Molecular Neurogenesis, RIKEN Brain Science Institute, Wako, Saitama, Japan; 3 Faculty of Science and Technology, Tokyo University of Science, Noda, Chiba, Japan; 4 Division of Neurobiology and Bioinformatics, National Institute for Physiological Sciences, Okazaki, Aichi, Japan; 5 Animal Immune and Cell Biology Research Unit, National Institute of Agrobiological Sciences, Tsukuba, Ibaraki, Japan; Universidade Federal do Rio de Janeiro, Brazil

## Abstract

Phospholipase D4 (PLD4) is a recently identified protein that is mainly expressed in the ionized calcium binding adapter molecule 1 (Iba1)-positive microglia in the early postnatal mouse cerebellar white matter. Unlike PLD1 and PLD2, PLD4 exhibits no enzymatic activity for conversion of phosphatidylcholine into choline and phosphatidic acid, and its function is completely unknown. In the present study, we examined the distribution of PLD4 in mouse cerebellar white matter during development and under pathological conditions. Immunohistochemical analysis revealed that PLD4 expression was associated with microglial activation under such two different circumstances. A primary cultured microglia and microglial cell line (MG6) showed that PLD4 was mainly present in the nucleus, except the nucleolus, and expression of PLD4 was upregulated by lipopolysaccharide (LPS) stimulation. In the analysis of phagocytosis of LPS-stimulated microglia, PLD4 was co-localized with phagosomes that contained BioParticles. Inhibition of PLD4 expression using PLD4 specific small interfering RNA (siRNA) in MG6 cells significantly reduced the ratio of phagocytotic cell numbers. These results suggest that the increased PLD4 in the activation process is involved in phagocytosis of activated microglia in the developmental stages and pathological conditions of white matter.

## Introduction

Phospholipase D4 (PLD4) is a member of the recently defined non-classical PLD family, which is characterized by two conserved HKD motifs (His-x-Lys-xxxx-Asp) in the C-terminal region [1]. In mammals, three additional members, Sam-9 [2] [now designated as PLD3 (MGI: 1333782)], PLD5 (MGI: 2442056), and mitoPLD [3] [now designated as PLD6 (MGI: 2687283)] have been identified in this family. HKD motifs are essential for PLD enzymatic activity [4], however, unlike the classical types PLD1 and PLD2, non-classical PLDs exhibit no typical PLD enzymatic activity for conversion of phosphatidylcholine into choline and phosphatidic acid [2], [5]. Furthermore, the members lack two functional domains, phox homology (PX) and pleckstrin homology (PH); both of which are found in the N-terminal regions of PLD1 and PLD2, and are involved in membrane targeting that leads to membrane localization and activation of PLD [6], [7], [8], [9], [10]. Instead, the non-classical PLD family is composed of a short N-terminal cytoplasmic tail, a transmembrane domain, and a relatively long C-terminal region [1]. In PLD4, nine consensus N-glycosylation sequences have been found in the C-terminal region and the molecular weight has been shifted down by deglycosylation, which suggests that this protein is a type II membrane glycoprotein. Although classical PLD1 and PLD2 are known to be involved in a variety of cellular functions, including intracellular transport, secretion, neuroprotection, phagocytosis, and cellular adhesion [11], [12], [13], [14], [15], probably by mediating phospholipid signaling, biological information of these novel PLD family members is still limited.

The expression of PLD4 is strictly regulated in mouse brain development. By RT-PCR analysis, PLD4 mRNA was first detected in mouse cerebellum at postnatal day 0 (P0), increased with age and peaked at P7, and then rapidly decreased to adult levels by P21 [1]. A double labeling study of P7 mouse cerebellum has shown that PLD4 mRNA is specifically present in ionized calcium binding adapter molecule 1 (Iba1)-positive microglia. It is well known that microglial activation occurs only for a short time at this stage of cerebellar development [16], therefore, PLD4 expression might be associated with activation of these cells. In addition to the brain, PLD4 mRNA has been detected in the mesenchymal organs, including thymus, liver, and spleen by GeneChip microarray analysis. In spite of its characteristic expression patterns, no information about its function is available to date.

In the present study, we investigated the role of PLD4 in microglia. We analyzed the distribution of PLD4 mRNA in mouse cerebellar white matter, during development and under pathological conditions, to determine whether PLD4 expression was associated with microglial activation. The function of PLD4 was examined using a primary cultured microglia and microglial cell line; both of which were derived from C57BL/6J mouse brain. Our results demonstrated that PLD4 expression was closely associated with microglial activation, and inhibition of its expression by siRNA led to a significant decrease in phagocytotic cells. This suggests that this protein is involved in phagocytosis of microglia in the central nervous system (CNS) under physiological and pathological conditions.

## Materials and Methods

### Animals

C57BL/6J mice were purchased from Japan SLC (Hamamatsu, Japan) and sacrificed at postnatal day (P) 0, 3, 5, 7, 10 and 21. The transgenic mouse line that contained two copies of mouse myelin proteolipid protein (PLP) gene [17] was maintained in the Animal Facility of the National Institute for Physiological Sciences. Heterozygote (*plp* ^tg/−^) mice at 4.5 months of age and their wild type littermates were used in this study. All animal experiments in this study were conducted according to recommendations and protocols approved by the Tokyo University of Pharmacy and Life Sciences Animal Use Committee (#Y09-15).

### Cells

For microglial culture, a mixed glial culture was prepared from cerebral cortices of 1-day-old C57BL/6J mice according to the method of Giulian and Baker (1986) [18]. After mechanical and chemical dissociation, the cells were cultured in Dulbecco's Modified Eagle's Medium (DMEM): nutrient mixture F12 (GIBCO/Invitrogen, Carlsbad, CA, USA) with 10% fetal bovine serum (FBS) (GIBCO/Invitrogen) at 37°C in 5% CO_2_. Medium was replaced every 4–5 days, and confluency was achieved after 14 days *in vitro*. Primary microglia were collected by gentle shaking by hand to prevent activation.

MG6 cell line is a c-myc-immortalized cell line of mouse microglia [19]. MG6 cells were maintained in 5% CO_2_ at 37°C in DMEM supplemented with 10% FBS, 10 µg/ml insulin (Sigma–Aldrich, Saint Louis, MO, USA) and 0.1 mM 2-mercaptoethanol (2-ME).

### Enzyme-linked immunosorbent assay (ELISA)

To determine the microglial activation levels during lipopolysaccharide (LPS) stimulation, the amount of tumor necrosis factor (TNF)-α in the culture medium was measured by mouse TNF-α instant ELISA kit, according to the manufacturer's instructions (Bender Med Systems, Vienna, Austria).

### Antibodies

The following antibodies were used for immunohistological and immunocytochemical studies: rabbit polyclonal anti-Iba1 antibody (1∶400) (Wako, Osaka, Japan); rat monoclonal anti-myelin basic protein (MBP) (1∶100) antibody (Chemicon/Millipore, Billerica, MA, USA); rabbit polyclonal anti-PLD4 C-terminal antibody (1∶200), which was produced by immunization with 16 amino acid residues (from amino acid 488 to 503, YAMDLDRQVPSQDCVW) of PLD4 [1]; rat monoclonal anti-CD11b antibody (1∶100) (AbD Serotec, Kidlington, UK); biotinylated anti-rabbit and anti-rat IgG antibodies (1∶250) (Vector Laboratories, Burlingame, CA, USA); and Alexa 350-, 488- and 594-conjugated anti-rabbit and anti-rat IgG antibodies (1∶2000) (Molecular Probes, Invitrogen).

### *In situ* hybridization

PLD4 cDNA (nucleotides 103–1613 corresponding to Genbank NM_178911) was cloned in pcDNA3 vector and used to prepare the probes. Digoxigenin-labeled antisense riboprobes were prepared using DIG RNA labeling kit (Roche, Basel, Switzerland). Paraffin sections of cerebella were prepared from P0–P21 C57BL/6J mice. The sections (6 µm) were treated with proteinase K (P0, 2 min; P3, 5 min; P5, 10 min; P7 and P10, 15 min; P21, 20 min), as described previously [20], [21]. The sections were hybridized with 2 µg/ml riboprobes at 60°C two overnight, and color development was achieved by incubation with nitroblue tetrazolium/5-bromo-4-chloro-3-indolyl-phosphate (Roche).

### Immunohistochemical analysis

The paraffin sections (6 µm) were boiled in citrate buffer (pH 6.0) for 1 min in a microwave oven for heat-induced antigen retrieval. The sections were incubated for 1 h in 0.01 M phosphate-buffered saline (PBS) that contained 0.3% Triton X-100 and 10% goat serum (PBS-TGS), and then overnight at 4°C with primary antibodies diluted in PBS-TGS. After rinsing, the sections were incubated with biotinylated secondary antibodies for 30 min at room temperature (RT). They were incubated with the ABC reagent (1∶50) (Vector Laboratories) for 30 min at RT, and immunoreactions were visualized using 0.005% H_2_O_2_ in 3,3′-diaminobenzidine/50 mM Tris buffer for 10 min at RT. Images were captured by light microscopy (Axio Scope Imaging System; Carl Zeiss, Oberkochen, Germany). For quantification, the cells were counted in three different regions (gray matter, proximal and distal white matter). Distal white matter indicated subcortical white matter. PLD4-positive cell numbers were obtained from six individual sections from mice at each age. The areas were measured by ImageGauge v4.23 (Fujifilm Tokyo Japan).

### Immunocytochemical analysis

Primary microglia were collected by gentle shaking by hand, and were transferred directly onto poly-L-lysine coated 13-mm cover slips overnight. A total of 10^5^ MG6 cells were grown on 13 mm cover slips overnight. The cells were treated with LPS (500 ng/ml) or vehicle (PBS) for 24 h. The cells were fixed with 4% paraformaldehyde on ice for 30 min, and preincubated for 1 h in PBS-TGS. The cells were incubated overnight at 4°C with primary antibodies diluted in PBS-TGS. The cells were then incubated with Alexa 350-, 488-, or 594-conjugated secondary antibodies for 1 h at RT. Images were captured by confocal microscopy (Olympus, Tokyo, Japan). Intensity of PLD4 signal per µm^2^ of each nucleus was measured by FV10-ASW v3.0 (Olympus).

### Western blot analysis

The MG6 cells were plated at 2.5×10^5^ cells on a 100-mm Petri dish. After cells became confluent, the plate was washed with PBS, and homogenization buffer that contained 0.32 M sucrose, 5 mM Tris–HCl, pH7.5, 0.75 µM aprotinin, 1 µM leupeptin, 1 µM pepstatin, 0.4 mM phenylmethylsulfonyl fluoride (PMSF), and 1 mM dithiothreitol (DTT) was added. MG6 cells were harvested by a cell scraper, and lysed by syringe with sequential passes through 21 G, 23 G and 26 G needles. For nuclear fractionation, hypotonic homogenization buffer that contained 0.5% Nonidet-P40, 10 mM HEPES (pH 7.9), 10 mM KCl, 1.5 mM MgCl_2_, 0.75 µM aprotinin, 1 µM leupeptin, 1 µM pepstatin, 0.4 mM PMSF, and 1 mM DTT was used. The nuclear fraction was collected as the pellet by centrifugation of whole cell homogenate at 8,000 rpm (MX-200; Tomy, Tokyo, Japan) for 15 min. The protein concentration was determined by the bicinchoninic acid method (Pierce/Thermo Scientific, Rockford, IL, USA). For deglycosylation, the samples were incubated at 95°C for 5 min in the presence of 0.5% sodium dodecyl sulfate (SDS) and 100 mM 2-ME, and cooled on ice for 3 min. Triton X-100 were added to a final concentration of 2.5%, and incubated with 0.25 U/30 µg protein of Peptide: N-glycosidase F (PNGase F) (Roche) for 16 h at 30°C.

Sample preparation, SDS-polyacrylamide gel electrophoresis (PAGE), and immunoblot analysis were performed as previously described [22]. MG6 cell homogenates were mixed with 2× sample buffer (0.25 M Tris–HCl, pH 6.8, 20% glycerol, 4% SDS, 0.2 M DTT, and bromophenol blue) and separated by SDS-PAGE (10.5%, 7×8 cm, 1 mm thick). The proteins were electrotransferred onto polyvinylidine difluoride membranes (Amersham/GE Healthcare, Piscataway, NJ, USA) at 30 V for 16 h at 4°C. For immunoblot analysis, anti-PLD4 antibody (1∶500) and horseradish-peroxidase-conjugated anti-rabbit IgG antibody (1∶5000) were used. The bands were detected using ECL western blot system (Amersham/GE Healthcare). For quantification, the intensity of each band was measured by ImageGauge v4.23, and the relative increase of PLD4 level induced by LPS stimulation was calculated. Values were obtained from four experiments.

### siRNA treatment

The commercially available double-stranded siRNA oligonucleotide against PLD4 gene was purchased from Invitrogen. The MG6 cells were transfected by 100 nM siRNA against PLD4 or control siRNA (Invitrogen) for 48 h with the PrimaPort siRNA transfection reagent (Credia-Japan, Kyoto, Japan), according to the manufacturer's protocol. The sequences of the double strand PLD4-siRNA sets were as follows:

sense siRNA; 5′-UGAUGUGGGAUGAGAAGUUCCGAGG-3′,

anti-sense siRNA; 5′-CCUCGGAACUUCUCAUCCCACAUCA-3′.

### RT-PCR analysis

Total RNA was extracted from MG6 cells grown in six-well tissue culture plates (7×10^4^ cells/well) by TRIzol Plus RNA Purification Kit (Invitrogen). Isolated total RNA was then reverse transcribed with TaKaRa RNA LA PCR™ Kit (AMV) Ver.1.1 (Takara Bio Inc., Shiga, Japan). PLD4 and control glyceraldehyde-3-phoshate dehydrogenase (GAPDH) cDNAs were amplified by the following specific primer sets:

PLD4 forward; 5′-GGGGGTGTTCTACACTCCAA-3′,

PLD4 reverse; 5′-GCACATGCACCCTTATTG-3′,

GAPDH forward: 5′-AATGGTGAAGGTCGGTGTGAAC-3′,

GAPDH reverse; 5′-GAAGATGGTGATGGGCTTCC-3′.

### Phagocytosis assay

Primary microglia were transferred directly onto cover slips and MG6 cells were plated at 10^5^ cells/well in six-well tissue culture plates that contained 1.5 ml DMEM/10% FBS. The cells were treated with LPS (500 ng/ml) or vehicle for 24 h. For simple phagocytosis assay, Alexa-594-conjugated *Escherichia coli* BioParticles (Molecular Probes/Invitrogen) were added at a concentration of 4×10^6^ BioParticles per well and left for 60 min. To determine the types of endosomes, the cells were incubated with transferrin–Alexa 594 (5 µg/ml) (Invitrogen) for 15 min or LysoTracker-DNRed99 (1 µM) (Invitrogen) for 30 min at 37°C. After washing, Alexa-488-conjugated *E. coli* BioParticles (Molecular Probes/Invitrogen) were added. After rinsing, the cells were immunostained by anti-PLD4 antibody as described. For quantification, PLD4^+^, PLD4^+^/lysotracker^+^, and PLD4^+^/transferrin^+^ vesicles per cell were counted in 20 randomly selected cells from each sample. Mean percentages of double-positive vesicles among total PLD4^+^ vesicles per cell were calculated.

For siRNA treatment, MG6 cells were plated at 7×10^4^ cells/well in six-well tissue culture plates. The cells were treated with PLD4-siRNA (100 nM), control-siRNA (100 nM) or vehicle for 48 h by PrimaPort siRNA transfection reagent. After rinsing, Alexa-488-conjugated *E. coli* BioParticles were added as described above. After rinsing, phagocytotic MG6 cells were analyzed by fluorescence-activated cell sorting (FACS) (Becton Dickinson).

### Cell proliferation assay

MG6 cells were plated at 5,000 cells/well in a 96-well microplate that contained 200 µl DMEM/10% FBS. They were treated with PLD4-siRNA (100 nM), control-siRNA (100 nM) or vehicle for 48 h. The following day, the cells were quantitated using a CCK-8 cell counting kit (Dojindo Laboratories, Kumamoto, Japan). After 2 h incubation with the reagent, absorbance at 460 nm was determined using a microplate reader (Tecan, Männedorf, Switzerland). The measured absorbance at 0 h after adding LPS or PBS was used as a standard value.

## Results

### Localization change of PLD4 mRNA-positive cells in developing mouse cerebellum

**Figure 1:**
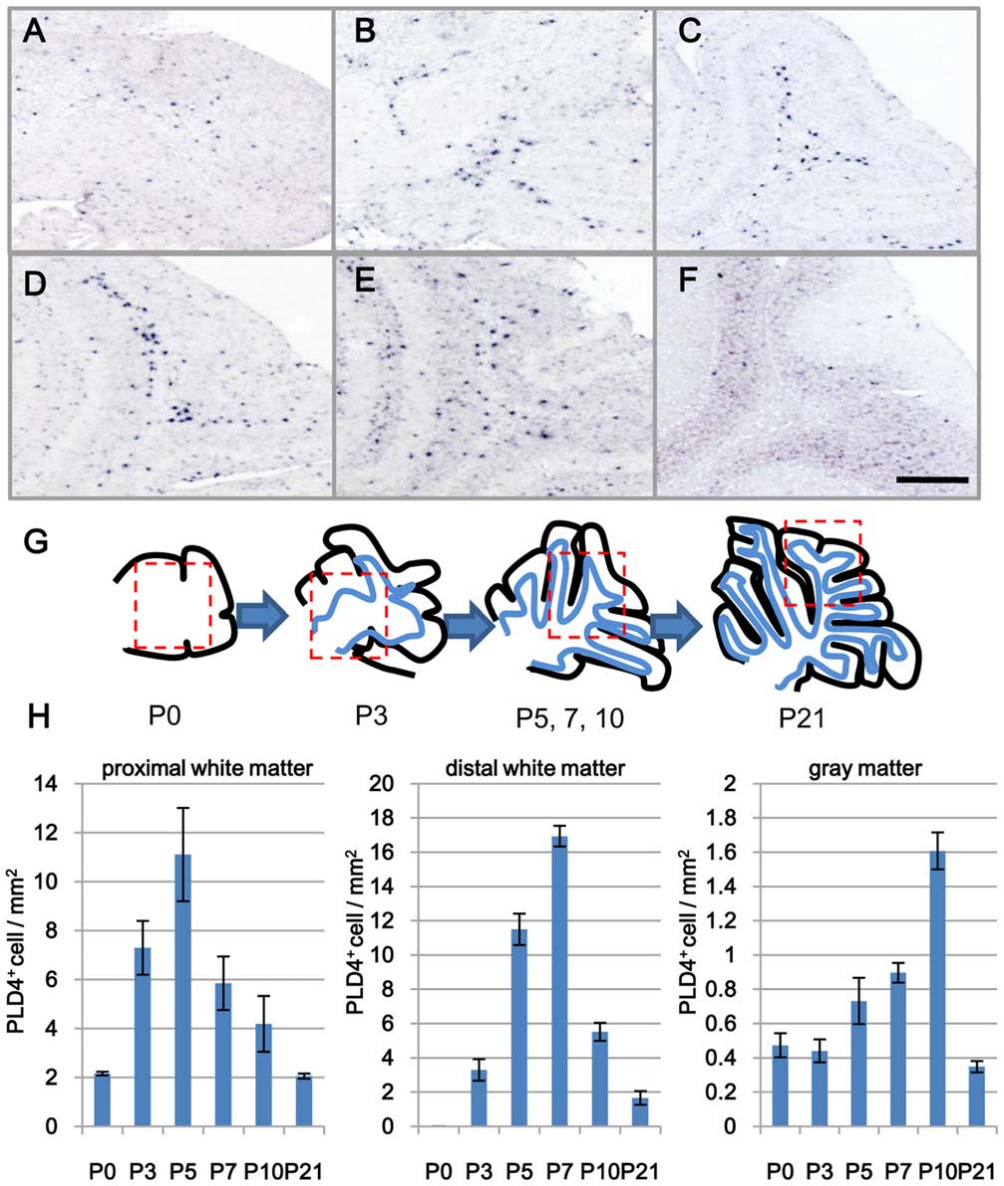
Localization of PLD4-mRNA-expressing cells in developing mouse cerebellum. Distribution of PLD4-mRNA-positive cells was examined by *in situ* hybridization in developing mouse cerebellum. PLD4-positive signals started to appear in the cerebellum at P0 (A). PLD4-positive cells increased in the proximal cerebellar white matter at P3 (B), and were distributed widely in the distal cerebellar white matter at P5 (C) and P7 (D). Signals started to be dispersed to the gray matter at P10 (Fig. 1E) and were rarely found at P21 (Fig. 1F). The morphology of cerebellar white matter during development is illustrated (G). The red dotted squares indicate the approximate fields of view. Scale bar, 100 µm. PLD4-positive cell numbers per area were obtained from six individual sections of mice at each age (H). The data were presented as mean ± SE of six experiments.

In our previous study, PLD4 mRNA was expressed in activated microglia in 7-day-old cerebellar white matter [1]. In order to examine the changes of distribution and numbers of PLD4-mRNA-expressing cells during development, *in situ* hybridization was performed with a specific probe for PLD4 mRNA in mouse cerebella at various developmental stages (Fig. 1). PLD4-positive signals were present only in a small number of cells inside of the cerebellum at P0 (Fig. 1A, H). PLD4-positive cell number increased in the proximal cerebellar white matter at P3 (Fig. 1B, H) and they were distributed widely in the distal cerebellar white matter at P5 and P7 (Fig. 1C, D, H). The signals became dispersed to gray matter at P10 (Fig. 1E, H) and were rarely found at P21 (Fig. 1F, H). These distribution patterns were well correlated to those of activated microglia in developing cerebellar white matter, as described previously [23]. This suggested that PLD4 expression was spatially and temporarily restricted during early postnatal development in mouse cerebellum.

### Increase of PLD4 expression in demyelinating lesions of *plp* ^tg/−^ mice

**Figure 2:**
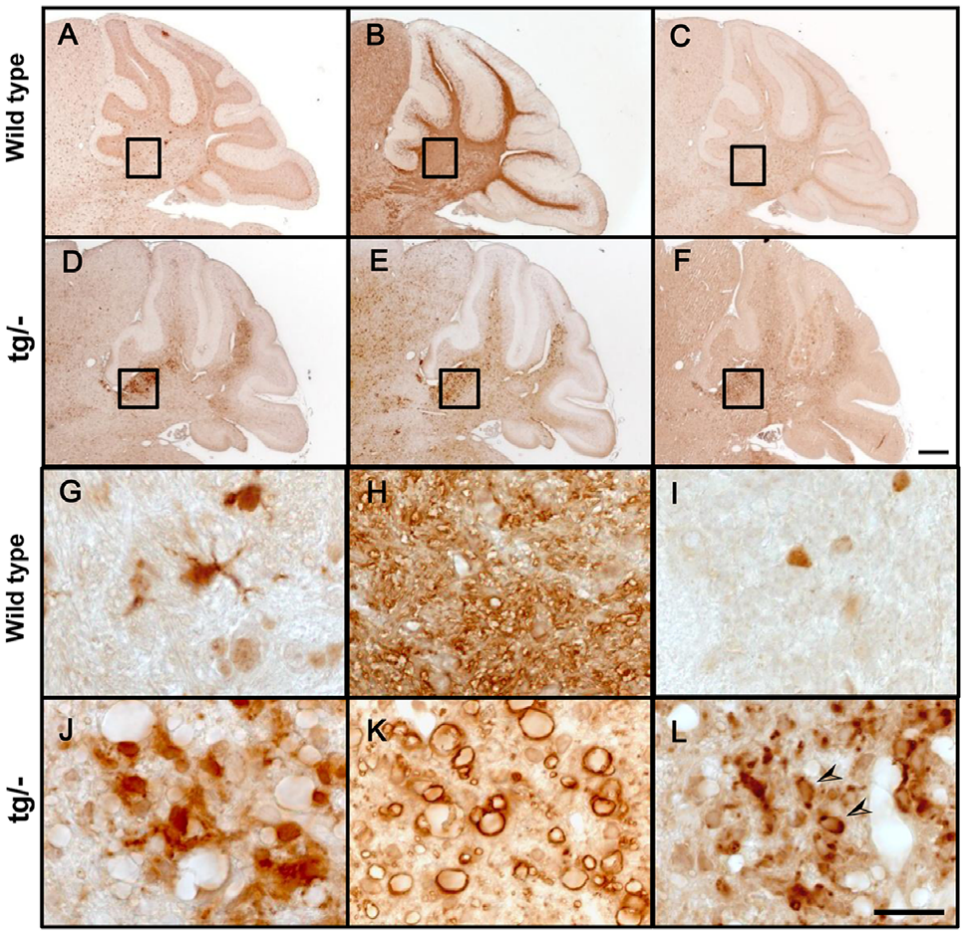
Expression of Iba1, MBP and PLD4 in the cerebella of PLP^tg/−^ and wild type mice. Sections prepared from 4.5-month-old PLP^tg/−^ (D–F) and wild type (A–C) mouse cerebella were immunostained with antibodies against Iba1 (A, D), MBP (B, E), and PLD4 (C, F). In wild type, weak signals of Iba1 (A) and PLD4 (B) were found in white matter. In contrast, the Iba1-positive (D) and PLD4-positive (F) strong signals were observed in the proximal cerebellar white matter of PLP^tg/−^ mice, where abnormal MBP-positive signals indicated demyelinated lesions (E). The enlarged images of the squares in A–F are shown in G–L, respectively. The results indicate that PLD4 is upregulated in activated microglia in demyelinating conditions in cerebellar white matter. Arrowheads in L show strong PLD4 signals in the cytoplasm. Scale bars; 100 µm in F for A–F and 20 µm in L for G–L.

Microglial activation occurs under various pathological conditions in adult brain. In order to clarify whether expression of PLD4 is induced in adult brains under pathological conditions that activate microglia, we investigated the expression of PLD4 in demyelinating lesions in the *plp* ^tg/−^ mice [17]. In this mutant, normal myelin was formed during development. After 2 months of age, degeneration of the CNS myelin becomes apparent although it was initially accompanied by remyelination until 6 months of age [17]. Sections of 4.5-month-old *plp* ^tg/−^ and wild type mouse cerebella were immunostained with antibodies against Iba1 (Fig. 2A, D), MBP (Fig. 2B, E), and PLD4 (Fig. 2C, F). In the *plp* ^tg/−^ mice, activated microglia showed intense signals of Iba1 and PLD4 (Fig. 2D, J and F, L) in the area where abnormal MBP signals were observed (Fig. 2E, K). The intense signal patterns for Iba1, MBP and PLD4 in *plp* ^tg/−^ mice were not observed in wild type littermates (Fig. 2A–C, G–I). The data indicated that PLD4 appeared in activated microglia in the demyelinating lesions of adult brain.

### Upregulation of PLD4 in the nuclei of activated microglia by LPS stimulation

**Figure 3:**
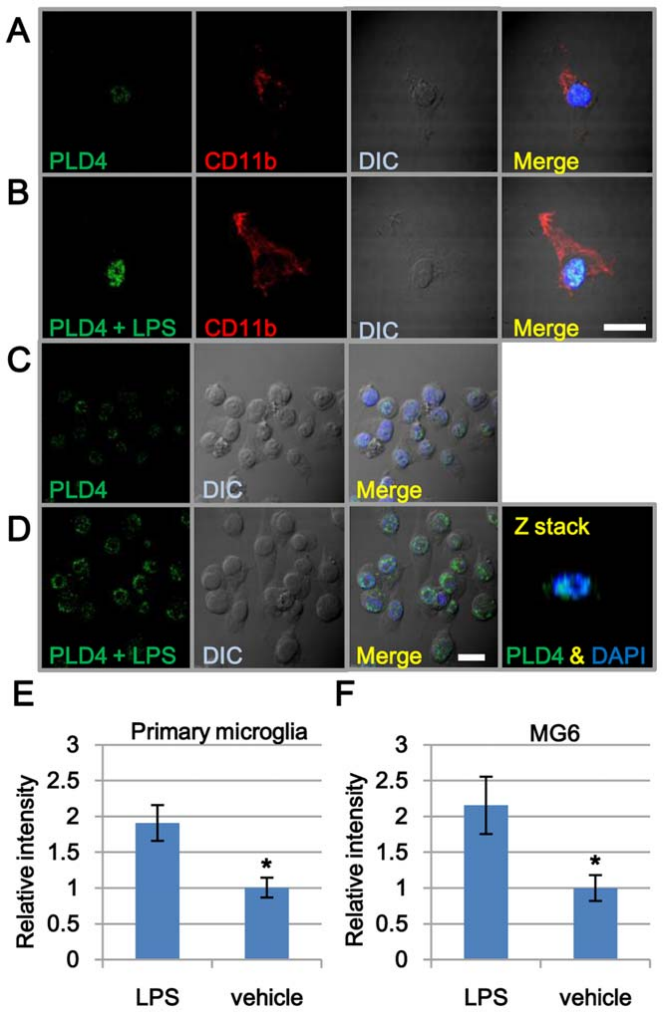
Nuclear localization of PLD4 in primary microglia and MG6 cells. Primary microglial cells (A, B) and MG6 cells (C, D) were treated with LPS (500 ng/ml) (B, D) or vehicle (A, C). (A and B) Primary cultured microglia were identified by CD11b-positive signals (red). The nuclei were weakly positive for PLD4 in vehicle-treated microglia (A), whereas strong PLD4 signals were found in the nuclei of LPS-stimulated microglia (B). (C and D) In MG6 cells, PLD4-positive signals (green) were detected in the nucleus, except the nucleoli (C). The signal intensities of PLD4 in the nuclei were markedly increased in LPS-stimulated MG6 cells (D). The Z-stack image of the nucleus was obtained using confocal microscopy. PLD4-positive signals (green) were detected in the internal part of the nucleus (blue) in MG6 cells (D). Blue signals indicated DAPI-stained nuclei. Scale bars, 20 µm. Intensity of nuclear PLD4 signals in LPS-treated primary microglia (E) and MG6 cells (F) was calculated against that of vehicle-treated controls. The data were presented as mean ± SE of four experiments. Asterisks in E and F indicate P<0.01 (Mann-Whitney's U test).

To determine the subcellular localization of PLD4 in microglia, we used a primary microglia derived from cerebral cortices of 1-day-old C57BL/6J mice. Primary microglia were cultured with or without LPS for 24 h, and immunocytochemical analysis was performed using anti-PLD4 and anti-CD11b antibodies. Only weak PLD4-positive signals (green) were detected in nuclei excluding the nucleolus of the vehicle-treated CD11b-positive (red) microglia (Fig. 3A). In contrast, the signal intensities of PLD4 in the nucleus were much higher in LPS-stimulated cells compared to those in the vehicle-treated cells (Fig. 3B, E). The nuclear localization of PLD4 was also found in the MG6 microglial cell line (Fig. 3C). PLD4-positive signal intensity was enhanced by 24 h LPS stimulation in MG6 cells (Fig. 3D, F). Dotted signals scattered in the cytoplasm became prominent after LPS treatment.

### PLD4 localization in phagosomes in phagocytic microglia

**Figure 4:**
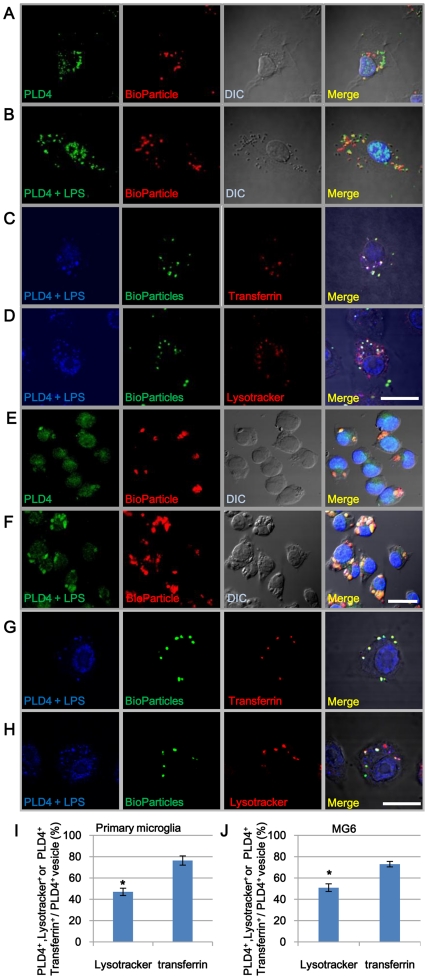
Localization of PLD4 in phagosomes of primary microglia and MG6 cells during phagocytosis. Primary cultured microglia (A, B) and MG6 cells (E, F) were treated with LPS (500 ng/ml) (B, F) or vehicle (A, E) and were incubated with BioParticles (red). The cells were immunostained with anti-PLD4 antibody (green). LPS treatment stimulated phagocytosis, and these cells contained more BioParticles (B, F). PLD4-positive signals were co-localized with BioParticles in phagosomes (yellow in A, B, E, F), whereas they were mainly found in the nuclei of the cells without BioParticles as shown in Fig. 3. LPS-stimulated primary cultured microglia (C, D) and MG6 cells (G, H) were incubated with BioParticles (green). The cells were double labeled with anti-PLD4 antibody (blue) and Alexa 594-transferrin or Lysotracker-DNRed99 (red). PLD4- and transferrin-positive signals were co-localized in BioParticle-containing phagosomes (white in C and G), whereas PLD4 and Lysotracker signals were co-localized only in a few phagosomes (D and H). Scale bar in F (for E and F), 20 µm. Scale bar in D (for A to D) and H (for G and H), 10 µm. Percentages of PLD4^+^/Lysotracker^+^ (Lysotracker) or PLD4^+^/Transferrin^+^ (transferrin) vesicles were compared in primary microglia (I) and in MG6 cells (J). The data were presented as mean ± SE of four experiments. Asterisks in I and J indicate P<0.01 (Mann-Whitney's U test).

As shown in Fig. 2L (arrowheads), strong PLD4 signal was observed in the cytoplasm of activated microglia in demyelinating area. Activated microglia have phagocytic activity. Difference of PLD4 localization between cultured cells and the cells in demyelinating area may be due to difference of activation levels of these cells. Therefore, the subcellular localization of PLD4 in primary microglial cells was examined during phagocytosis of fluorescent BioParticles. After microglia were maintained with (Fig. 4B) or without (Fig. 4A) LPS for 24 h, the cells were incubated further with BioParticles for 1 h. PLD4-positive signals (green) were found in BioParticle-containing phagosomes (yellow in merged pictures in Fig. 4A, B). These data suggest the involvement of PLD4 in phagocytosis.

To establish whether PLD4 was implicated in early or late endosomes, primary microglial cells were labeled with transferrin (early endosome marker) or lysotracker (late endosome maker), and PLD4 localization was examined after phagocytosis of BioParticles. Most PLD4-positive signals were found in transferrin-containing phagosomes (Fig. 4C), whereas fewer signals were overlapped with lysotracker-positive phagosomes (Fig. 4D). Also, no PLD4-positive signals were observed in the plasma membrane. The results suggest that PLD4 is predominantly present in early phagosomes (Fig. 4I). The same results were obtained by the experiments using MG6 cells (Fig. 4E, F, G, H and J). Thus, upregulation and subcellular localization of PLD4 in activated MG6 cells is identical to that in activated primary microglia. Therefore, we used MG6 cells for further studies to establish a functional role of PLD4.

### Increase of PLD4 protein level by LPS stimulation in MG6 cells

**Figure 5:**
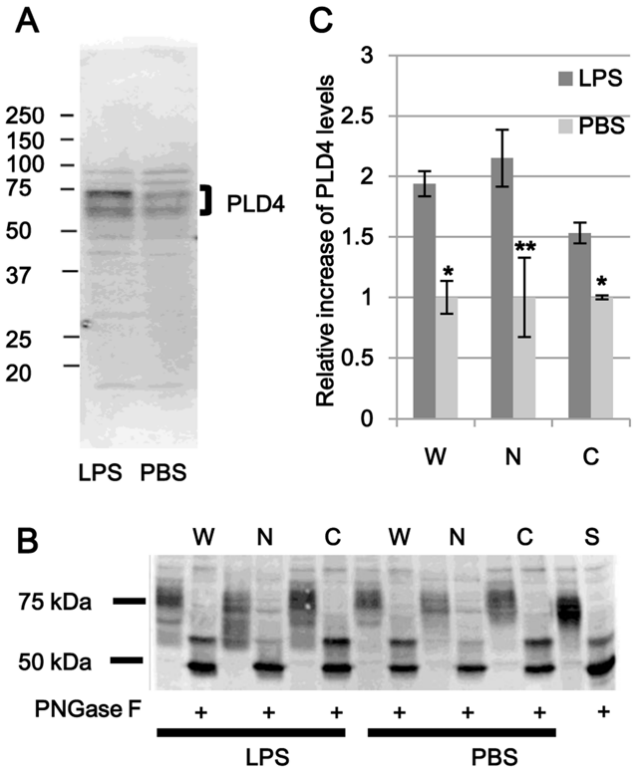
Increase of PLD4 level by LPS stimulation in MG6 cells. MG6 cells were treated with LPS (500 ng/ml) or vehicle (PBS) for 24 hrs. (A) The Western blot analysis (15 µg proteins, 10.5% SDS-PAGE) using anti-PLD4 antibody revealed that the levels of PLD4-related bands (70–80 kDa) were increased in LPS-stimulated MG6 cells (left) compared with those in control cells (right). (B) Whole cell homogenates (W), nuclear fractions (N), and supernatants (C) were prepared. Mouse spleen homogenate (S) was prepared as a positive control. Each sample was deglycosylated by peptide-N-glycosidase F (PNGase F). Western blot analysis (15 µg proteins, 10.5% SDS-PAGE) using PLD4 antibody revealed that PLD4-positive bands were found in the nuclear fraction and supernatants. After PNGase F treatment, PLD4-positive band sizes were changed (48 kDa). (C) Intensity of each band was measured and relative increase of PLD4 by LPS-stimulation in each fraction was expressed graphically. The data are presented as mean ± SE of four experiments. Asterisk and double asterisk in C indicate P<0.01 and P<0.05 by Mann-Whitney's U test, respectively. Levels of PLD4 were increased in all LPS-stimulated MG6 cell samples compared with those in control cell samples.

Increased level of PLD4 was confirmed by western blot using activated MG6 cells. After treatments of MG6 with LPS (500 ng/ml) or vehicle for 24 h, the secretion levels of TNF-α in the culture medium were 2,069±245 pg/ml in LPS-stimulated cells and 28±10 pg/ml in vehicle-treated control cells, which indicated that MG6 cells were activated. Western blot analysis of these cell homogenates exhibited multiple PLD4-related bands of 70–80 kDa (Fig. 5A). The multiple immunoreactive bands seemed to contain different sizes of glycosylated moieties [1] and their specificity was confirmed by antigen absorption test (data not shown). A marked increase in PLD4 bands was found in LPS-stimulated cells compared with controls (Fig. 5A). Thus, PLD4 expression was enhanced in LPS-activated MG6 cells.

The nuclear localization of PLD4 was also confirmed by western blot analyses of the fractions prepared from MG6 cells with or without LPS stimulation (Fig. 5B). Enrichment of nucleoporin and elimination of GRP78/Bip (endoplasmic reticulum fraction), COXIV (mitochondrial fraction) and GAPDH (cytosolic fraction) demonstrated the purity of nuclear fractions (data not shown). Spleen homogenate prepared from adult mice was used as a positive control. Immunoblots exhibited ladder-like bands, probably due to different levels of glycosylation at nine possible sites in PLD4 molecule; therefore, PLD4 levels in deglycosylated samples using PNGase F were compared. Two PLD4-related bands were confirmed by absorption tests (data not shown), and were detected in all the deglycosylated samples (Fig. 5B). The predicted size of PLD4 peptide was approximately 48 kDa, which suggests that the lower band represented completely deglycosylated PLD4. PLD4 was found in the nuclear fraction and the supernatants, and the amounts significantly increased in LPS-stimulated cells in all fractions (Fig. 5C). Approximately twofold increase in PLD4 level was observed in the nuclear fraction by LPS treatment.

### Inhibition of phagocytosis by PLD4 siRNA

**Figure 6:**
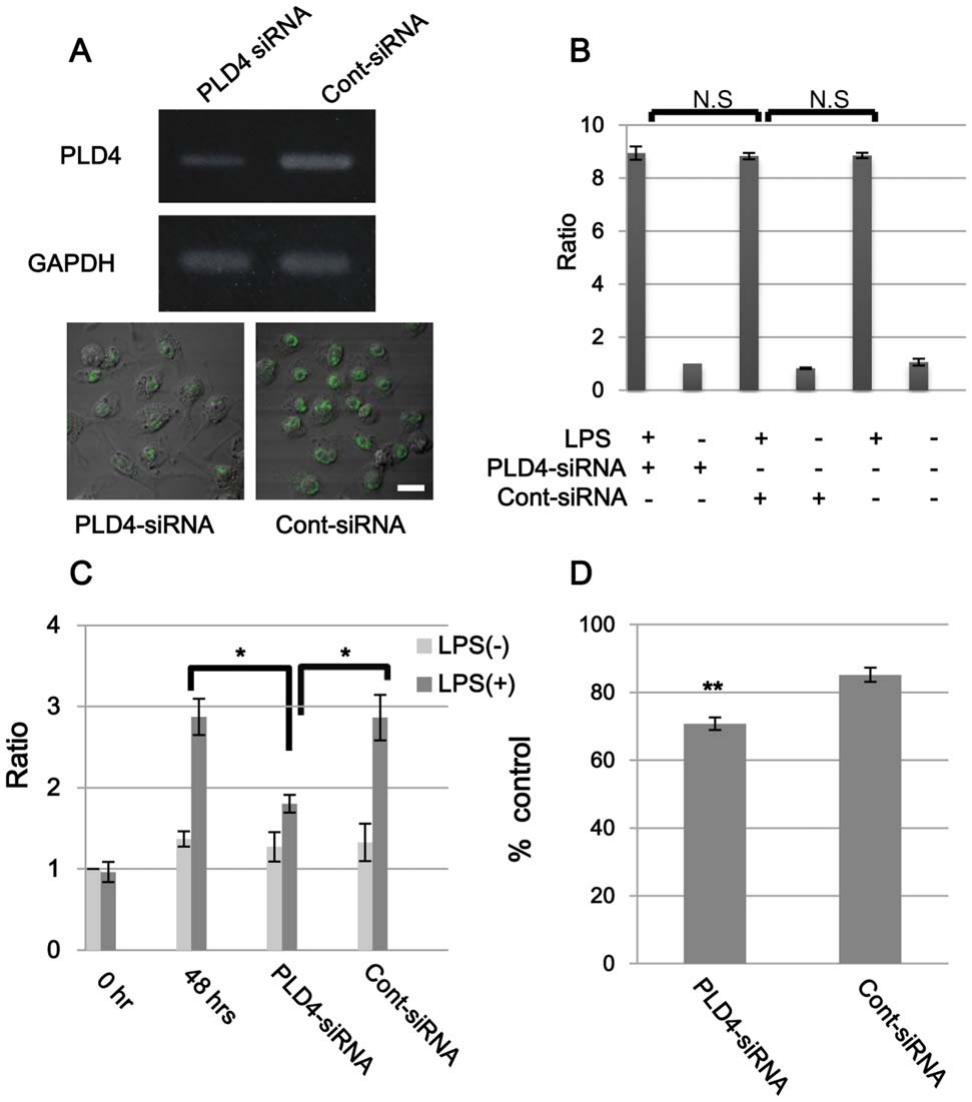
Influence of PLD4 knock down in MG6 cells by siRNA treatment. (A) MG6 cells were transfected with PLD4-siRNA or control (cont)-siRNA (100 nM each) for 48 h. Total RNA was isolated from these cells and analyzed by RT-PCR for PLD4 (above) and GAPDH (below) mRNA expression. Expression of PLD4 mRNA was efficiently reduced by 100 nM PLD4 siRNA treatment. The immunofluorescence staining of PLD4 (green) revealed that PLD4 was downregulated in PLD4-siRNA-treated cells (left), compared with Cont-siRNA (right) by 100 nM siRNA treatment. Scale bars, 20 µm. (B) MG6 cells were transfected with siRNA for 24 h, and LPS (500 ng/ml) or PBS (vehicle) were added to the medium. After 24 h, secretion of TNF-α was measured by ELISA. Measurement of PLD4-siRNA-treated cells was used as a standard value. Secretion of TNF-α was not significant in the siRNA-treated groups (n = 4). (C) LPS-stimulated (dark gray bars) or vehicle-treated (gray bars) MG6 cells were treated with or without siRNA for 48 h. Measurement at 0 h before addition of LPS or PBS was used as a standard value. Proliferation was examined by cell counting kit (n = 4). (D) MG6 cells were transfected with PLD4- or control (cont)-siRNA for 48 h. Vehicle-treated cells were used as a control. Cells were incubated with BioParticles. BioParticle-containing cells were analyzed by FACS, and phagocytic activity was calculated by dividing these cell numbers by the total. The graph shows the percentage of phagocytic activity of each siRNA-treated cells compared with that of the control cells. The data are presented as mean ± SE of five experiments. Asterisks in C and double asterisk in D indicate P<0.01 and P<0.05 by Mann-Whitney's U test, respectively.

Involvement of PLD4 in phagocytosis was examined by measuring phagocytotic activity under PLD4 knockdown conditions using RNA interference (RNAi). First, we searched for an appropriate RNAi condition, by which PLD4-siRNA showed the highest efficiency of inhibition of PLD4 expression in the MG6 cells. MG6 cells were transfected by PLD4- or control-siRNA for 48 h and PLD4 mRNA expression levels were examined. RT-PCR showed that MG6 cells silenced by 100 nM PLD4-siRNA exhibited a significantly higher rate of inhibition compared with cells treated by control-siRNA (Fig. 6A). Therefore, 100 nM of each siRNA was used for further studies. No apparent effects of PLD4 knockdown on TNF-α secretion were observed with and without LPS (Fig. 6B). LPS treatment of MG6 cells stimulated proliferation and the cell count increased about three times compared with the absence of LPS stimulation (Fig. 6C). However, the increase in cell numbers was significantly less when the cells were treated with PLD4-siRNA (Fig. 6C), whereas it was not significantly changed in cells that were not stimulated by LPS, regardless of PLD4 knockdown. These results suggest that loss of PLD4 did not affect microglial activation but inhibited the increase in cell numbers induced by LPS stimulation. After siRNA treatment followed by 1 h incubation with BioParticles, fluorescence-containing MG6 cells were counted by FACS. The ratio of phagocytotic cell number in each siRNA-treated group was compared with that in the control group (no siRNA) and the percentage was calculated. As shown in Fig. 6D, phagocytic activity in PLD4 siRNA-treated group (70.78±4.73%) was significantly decreased compared with that in control siRNA-treated group (85.21±5.48%, P<0.05). Taken together, these results suggest that PLD4 is involved in phagocytic potency of microglia-like MG6 cells.

## Discussion

**Figure 7:**
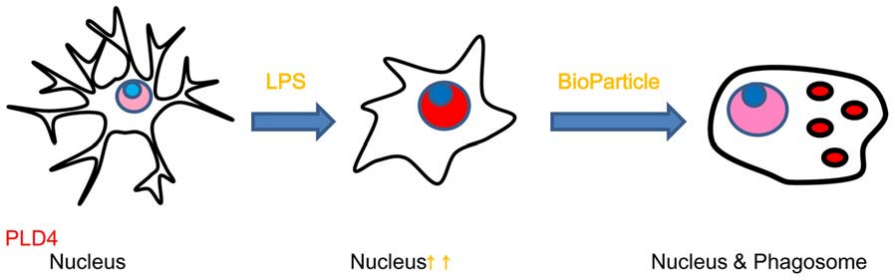
Localization change of PLD4 in cultured microglia. PLD4 was located in the nucleus during the resting state. After LPS stimulation, expression of PLD4 was increased. In the phagocytotic state eating BioParticles, PLD4 accumulated in the early phagosomes.

We demonstrated that one of the novel PLD family members containing the transmembrane domain, PLD4, was expressed in activated microglia found in developing cerebellum, as well as in demyelinated white matter lesions. Primary cultured microglia and the microglial cell line MG6 showed that PLD4 immuno-signals were mainly present in the nucleus, apart from the nucleolus, and were upregulated by LPS stimulation. PLD4 immunoreactivity accumulated in phagosomes when BioParticles were added to primary microglial cultures (Fig. 7). Inhibition of PLD4 expression significantly reduced the number of phagocytotic cells. Together, these results suggest that PLD4 is involved in phagocytosis of activated microglia. The present study is believed to be the first to demonstrate a role for one of the non-classical PLD family members in cell functions.

### PLD4 is upregulated in microglial activation by various stimuli

Microglia are cells of myeloid origin that are distributed diffusely throughout the brain. Microglia under normal conditions monitor the status of the local surroundings, whereas under pathological conditions, they migrate to the lesion, release a wide range of soluble factors that include cytotoxins, neurotrophins and immunomodulatory factors, and clear cellular debris by phagocytosis [24]. Microglia are also activated temporally in developing cerebellar white matter regions [16] where the projection of climbing fibers change from multiple to mono-innervation of Purkinje cells [25]. Under these conditions, microglia are stimulated by various humoral factors, as well as by direct contact of pathogens and cell debris. Gene expression patterns are different depending on the stimuli. One of the exogenous stimulating factors is LPS. LPS binds to Toll-like receptors and activates microglia by intracellular signaling pathways related to nuclear factor-κB and microtubule-associated protein kinase [26]. Binding of antibodies to Fc receptors on the microglial surface induces phagocytosis activity in microglia, which is associated with inflammation [27]. In contrast, signals mediated by TREM2 (triggering receptor expressed on myeloid cells-2) and the phosphatidylserine receptor are involved in microglial phagocytosis without inflammation, for example, of apoptotic cells [24]. In our *in vitro* and *in vivo* studies, PLD4 expression was upregulated by LPS treatment, demyelination and normal developmental processes. Thus, PLD4 function is probably associated with microglial activation via a wide variety of cell stimuli.

### Localization of PLD4 in microglia is dependent on their activation levels

In the adult CNS, microglia thought to have at least three clearly identifiable states, resting, reactive but non-phagocytic, and phagocytic [28]. PLD4 changes its expression level and its subcellular localization from the nucleus to phagosomes by activation of microglia. This change between reactive and phagocytic states is also observed in microglial cell lines, which suggests that it is a feature of this protein in microglia. In addition, accumulation of PLD4 in microglial cytoplasm in demyelinated lesions (Fig. 2L) implies the involvement of these microglial cells in phagocytic clearance, for example, removal of damaged myelin. At present, several markers for activated microglia are available, including Iba1 and CD11b. PLD4 is also a good marker for the state of the microglia, because its change of expression level and subcellular location are well correlated with microglial activation.

The distribution was different from our previous study of PLD4-transfected cells. When PLD4 is exogenously overexpressed in HEK293 or HeLa cells, the immunoreactivity is mainly observed in the Golgi complex and endoplasmic reticulum [1]. It is possible that only endogenous PLD4 protein can be transported into the nucleus of microglia in culture. Based on the nucleotide sequence (GenBank: NM_178911), PLD4 has a transmembrane domain, and regular nuclear localization signals are not found. Recent studies have demonstrated that certain membrane proteins, including fibroblast growth factor receptor [29], epidermal growth factor receptor [30]–[32] and ErbB-2 [33], are transported into the nucleus by the nuclear transport system mediated by importin β. Therefore, PLD4 might be transported by similar mechanisms. RNAi showed partial inhibition of proliferation during LPS stimulation, whereas the basic proliferation rate was not affected. This suggests its involvement in proliferation, although it is still possible that a decrease in PLD4 affects microglial activation level, and in turn, influences proliferation rate. Studies on PLD4 function in the nucleus are in progress.

### Classical and novel types of PLD family members are involved in phagocytosis

The classical PLDs are involved in phagocytosis of macrophages [34]. PLD1 is present in vesicles identified as the late endosomal/lysosomal compartment, whereas PLD2 is localized at the plasma membrane in macrophages [11], [35], [36]. In the phagocytic state of macrophages, PLD1-positive vesicles are recruited to nascent and internalized phagosomes, whereas PLD2 is only observed in nascent phagosomes. Double knockdown studies using specific siRNA against PLD1 and PLD2 have exhibited a decrease in the phagocytic rates to approximately 70% [11]. Thus, the classical PLD members play important roles in distinct processes of phagocytosis, probably through local production of phosphatidic acid. In contrast to the classical PLD family, PLD4 showed no enzymatic activity for hydrolysis of phosphatidylcholine to choline and phosphatidic acid [1]. Furthermore, a relatively long C-terminal tail with sugar moieties of PLD4 is probably located inside the phagosomes; therefore, the topology of the protein is different from that of classical PLDs. PLD4 is also expressed in the reticuloendothelial system [1]; therefore, understanding the mechanism of this protein could give us fundamental information about phagocytotic processes.

## References

[1] Yoshikawa F, Banno Y, Otani Y, Yamaguchi Y, Nagakura-Takagi Y (2010). Phospholipase D family member 4, a transmembrane glycoprotein with no phospholipase D activity, expressed in spleen and early postnatal microglia.. PLoS One.

[2] Pedersen KM, Finsen B, Celis JE, Jensen NA (1998). Expression of a novel murine phospholipase D homolog coincides with late neuronal development in the forebrain.. J Biol Chem.

[3] Choi SY, Huang P, Jenkins GM, Chan DC, Schiller J (2006). A common lipid links Mfn-mediated mitochondrial fusion and SNARE-regulated exocytosis.. Nat Cell Biol.

[4] Sung TC, Roper RL, Zhang Y, Rudge SA, Temel R (1997). Mutagenesis of phospholipase D defines a superfamily including a trans-Golgi viral protein required for poxvirus pathogenicity.. EMBO.

[5] Munck A, Bohm C, Seibel NM, Hashemol HZ, Hampe W (2005). Hu-K4 is a ubiquitously expressed type 2 transmembrane protein associated with the endoplasmic reticulum.. FEBS J.

[6] Du G, Altshuller YM, Vitale N, Huang P, Chasserot-Golaz S (2003). Regulation of phospholipase D1 subcellular cycling through coordination of multiple membrane association motifs.. J Cell Biol.

[7] Hodgkin MN, Masson MR, Powner D, Saqib KM, Ponting CP (2000). Phospholipase D regulation and localization is dependent upon a phosphatidylinositol 4,5-biphosphate-specific PH domain.. Curr Biol.

[8] Sciorra VA, Rudge SA, Prestwich GD, Frohman MA, Engebrecht J (1999). Identification of a phosphoinositide binding motif that mediates activation of mammalian and yeast phospholipase D isoenzymes.. EMBO J.

[9] Sciorra VA, Rudge SA, Wang J, McLaughlin S, Engebrecht J (2002). Dual role for phosphoinositides in regulation of yeast and mammalian phospholipase D enzymes.. J Cell Biol.

[10] Stahelin RV, Ananthanarayanan B, Blatner NR, Singh S, Bruzik KS (2004). Mechanism of membrane binding of the phospholipase D1 PX domain.. J Biol Chem.

[11] Corrotte M, Chasserot-Golaz S, Huang P, Du G, Ktistakis NT (2006). Dynamics and function of phospholipase D and phosphatidic acid during phagocytosis.. Traffic.

[12] Gomez-Cambronero J, Di Fulvio M, Knapek K (2007). Understanding phospholipase D (PLD) using leukocytes: PLD involvement in cell chemotaxis and adhesion.. J Leukoc Biol.

[13] Iyer SS, Agrawal RS, Thompson CR, Thompson S, Barton JA (2006). Phospholipase D1 regulates phagocyte adhesion.. J Immunol.

[14] Lee MY, Kim SY, Min DS, Choi YS, Shin SL (2000). Upregulation of phospholipase D in astrocytes in response to transient forebrain ischemia.. GLIA.

[15] Min do S, Choi JS, Kim HY, Shin MK, Kim MK (2007). Ischemic preconditioning upregulates expression of phospholipase D2 in the rat hippocampus.. Acta Neuropathol.

[16] Ashwell K (1990). Microglia and cell death in the developing mouse cerebellum.. Brain Res Dev Brain Res.

[17] Kagawa T, Ikenaka K, Inoue Y, Kuriyama S, Tsujii T (1994). Glial cell degeneration and hypomyelination caused by overexpression of myelin proteolipid protein gene.. Neuron.

[18] Giulian D, Baker TJ (1986). Characterization of ameboid microglia isolated from developing mammalian brain.. J Neurosci.

[19] Takenouchi T, Ogihara K, Sato M, Kitani H (2005). Inhibitory effects of U73122 and U73343 on Ca2+ influx and pore formation induced by the activation of P2X7 nucleotide receptors in mouse microglial cell line.. Biochim Biophys Acta.

[20] Sato A, Sekine Y, Saruta C, Nishibe H, Morita N (2008). Cerebellar development transcriptome database (CDT-DB): profiling of spatio-temporal gene expression during the postnatal development of mouse cerebellum.. Neural Netw.

[21] Yoshikawa F, Sato Y, Tohyama K, Akagi T, Hashikawa T (2008). Opalin, a transmembrane sialylglycoprotein located in the central nervous system myelin paranodal loop membrane.. J Biol Chem.

[22] Yamaguchi Y, Miyagi Y, Baba H (2008). Two-dimentional electropholesis with cationic detergent, a powerful tool for the proteomic analysis of myelin proteins. Part 1: Technical aspects of electropholesis.. J Neurosci Res.

[23] Wolswijk G (1995). Strongly GD3+ cells in the developing and adult rat cerebellum belong to the microglial lineage rather than to the oligodendrocyte lineage.. Glia.

[24] Walter L, Neumann H (2009). Role of microglia in neuronal degeneration and regeneration.. Semin Immunopathol.

[25] Hashimoto K, Kano M (2005). Postnatal development and synapse elimination of climbing fiber to Purkinje cell projection in the cerebellum.. Neurosci Res.

[26] Pyo H, Jou I, Jung S, Hong S, Joe EH (1998). Mitogen-activated protein kinases activated by lipopolysaccharide and beta-amyloid in cultured rat microglia.. Neuroreport.

[27] Okun E, Mattson MP, Arumugam TV (2010). Involvement of Fc receptors in disorders of the central nervous system.. Neuromol Med.

[28] Streit WJ, Kettenmann H, Ransom BR (1995). Microglial cells.. Neuroglia.

[29] Reilly JF, Maher PA (2001). Importin beta-mediated nuclear import of fibroblast growth factor receptor: role in cell proliferation.. J Cell Biol.

[30] Lin SY, Makino K, Xia W, Matin A, Wen Y (2001). Nuclear localization of EGF receptor and its potential new role as a transcription factor.. Nat Cell Biol.

[31] Lo HW, Ali-Seyed M, Wu Y, Bartholomeusz G, Hsu SC (2006). Nuclear-cytoplasmic transport of EGFR involves receptor endocytosis, importin b1 and CRM1.. J Cell Biochem.

[32] Wang YN, Yamaguchi H, Huo L, Du Y, Lee HJ (2010). The translocon Sec61b localized in the inner nuclear membrane transports membrane-embedded EGF receptor to the nucleus.. J Biol Chem.

[33] Giri DK, Ali-Seyed M, Li LY, Lee DF, Ling P (2005). Endosomal transport of ErbB-2: mechanism for nuclear entry of the cell surface receptor.. Mol Cell Biol.

[34] Kusner DJ, Hall CF, Jackson S (1999). Fc gamma receptor-mediated activation of phospholipase D regulates macrophage phagocytosis of IgG-opsonized particles.. J Immunol.

[35] Arneson LS, Kunz J, Anderson RA, Traub LM (1999). Coupled inositide phosphorylation and phospholipase D activation initiates clathrin-coat assembly on lysosomes.. J Biol Chem.

[36] Du G, Huang P, Liang BT, Frohman MA (2004). Phospholipase D2 localizes to the plasma membrane and regulates angiotensin II receptor endocytosis.. Mol Biol Cell.

